# Design of Optimal Rainfall Monitoring Network Using Radar and Road Networks

**DOI:** 10.3390/e23030378

**Published:** 2021-03-23

**Authors:** Taeyong Kwon, Seongsim Yoon, Sanghoo Yoon

**Affiliations:** 1 Department of Statistics, Daegu University, Gyeongbuk 38453, Korea; td1022@daegu.ac.kr; 2 Korea Institute of Civil Engineering and Building Technology, Goyang 10223, Korea; ssyoon@kict.re.kr; 3 Division of Mathematics and Big Data Science, Daegu University, Gyeongbuk 38453, Korea

**Keywords:** entropy, optimal rainfall network, precipitation

## Abstract

Uncertainty in the rainfall network can lead to mistakes in dam operation. Sudden increases in dam water levels due to rainfall uncertainty are a high disaster risk. In order to prevent these losses, it is necessary to configure an appropriate rainfall network that can effectively reflect the characteristics of the watershed. In this study, conditional entropy was used to calculate the uncertainty of the watershed using rainfall and radar data observed from 2018 to 2019 in the Goesan Dam and Hwacheon Dam watersheds. The results identified radar data suitable for the characteristics of the watershed and proposed a site for an additional rainfall gauge. It is also necessary to select the location of the additional rainfall gauged by limiting the points where smooth movement and installation, for example crossing national borders, are difficult. The proposed site emphasized accessibility and usability by leveraging road information and selecting a radar grid near the road. As a practice result, the uncertainty of precipitation in the Goesan and Hwacheon Dam watersheds could be decreased by 70.0% and 67.9%, respectively, when four and three additional gauge sites were installed without any restriction. When these were installed near to the road, with five and four additional gauge sites, the uncertainty in the Goesan Dam and Hwacheon Dam watersheds were reduced by up to 71.1%. Therefore, due to the high degree of uncertainty, it is necessary to measure precipitation. The operation of the rainfall gauge can provide a smooth site and configure an appropriate monitoring network.

## 1. Introduction

With the recent increase in extreme rainfall events such as localized heavy rainfall and flash floods due to the influence of climate change, water resource management has become more complicated and challenging. For efficient water resource management, accurate quantitative rainfall estimation is required. If the watershed of a reservoir is small, or if part of the watershed is situated in a neighboring country, rainfall estimation in the watershed becomes difficult because rainfall stations can be operated only within a limited area. This spatial restriction may be overcome with the use of radar data; quantitative precipitation estimation over a wide area can be achieved through radar reflectivity measurements. However, although quantitative precipitation estimated using radar data demonstrates a high correlation with measured precipitation, the two sets of values are not in exact agreement; therefore, more rainfall stations need to be installed for a more accurate quantitative estimation of rainfall.

The concept of entropy is widely used in the design of rainfall monitoring networks. The study [1] reported the usefulness and wide range of applicability of the entropy theory in hydrology. The uncertainty of rainfall has also been evaluated using the entropy of radar data [2,3,4]. In hydrology, entropy is also used in the evaluation and design of rain gauge networks [5,6], and in the evaluation of water quality monitoring networks [7]. Previous studies that applied entropy to rainfall observation data are as follows. The study [8] estimated the rainfall risk threshold, and the study [9] evaluated rainfall monitoring networks in areas with insufficient water resources. The study [10] designed a hydrometric network of watersheds using the concept of marginal entropy, joint entropy and transinformation. The studies [11,12,13,14] evaluated rainfall monitoring networks and presented the optimal network in terms of network composition. The design of an optimal rainfall monitoring network considering altitude as well as entropy was also investigated [15,16,17]. In addition, there have been studies on entropy approximating the nonparametric multivariate probability density function [18], rainfall characteristics based on temporal resolution, and correlation coefficients [19].

This study aims to investigate methods to identify locations for the installation of additional rainfall stations in areas where the watershed is small or where part of the watershed lies in a neighboring country. The Goesan Dam watershed has a high risk of overflow due to its small watershed area and low flood control storage capacity of the reservoir. For the Hwacheon Dam watershed, it is difficult to identify the characteristics of rainfall upstream of the catchment area because most of the area upstream of the dam—which includes North Korea as well—is ungauged. The data used for this study are rainfall observation data from the catchment areas of Goesan Dam and Hwacheon Dam, and data from radars capable of measurements over a wide range. In addition, road network data were considered for the determination of the location of additional rainfall stations that may be installed and managed effectively for better rainfall monitoring. In the existing research, the entropy was calculated for the entire radar grid to design the rainfall network. These methods recommend that the rainfall network is configured even in areas where the altitude is high or the approach is difficult. Using the road network to select only the radar grid near to the road, and calculating the entropy, enhances the access and operability that comprise the rainfall network.

The remainder of this paper is organized as follows. Section 2 discusses the entropy theory as a research methodology while Section 3 describes the study area, radar data, and road network data used in the study. Section 4 proposes a method for the design of an optimal rainfall monitoring network with additional rainfall stations in the Goesan Dam and Hwacheon Dam watersheds, based on entropy. Section 5 summarizes the research results and presents the limitations of this study as well as future directions the study may take.

## 2. Methods

This study proposes a method to identify locations of additional rainfall stations when there is difficulty in quantitative rainfall estimation in a watershed with a limited number of rainfall stations. The importance of rainfall monitoring networks can be evaluated by marginal entropy and conditional entropy [9]. 

Entropy is a quantitative measure of the degree of randomness in a probability distribution of data [20], while marginal entropy is a measure of uncertainty of a discrete random variable *X*, and is calculated by the following equation:

(1) $$H\left(X\right)=-\overset{N}{\underset{n=1}{\sum }}p\left(x_{n}\right)\ln p\left(x_{n}\right)$$

where H(X) is the marginal entropy representing the uncertainty of X, n is the number of class intervals of X, and $p\left(x_{n}\right)$ is the empirical probability of $x_{n}$. Rainfall stations with high entropy have a high degree of uncertainty in rainfall, and rainfall observation data are therefore highly important. The uncertainty of an additional station can be obtained using conditional entropy. Given the variable Y, the entropy of the variable X is calculated as follows:

(2) $$H\left(X|Y\right)=-\overset{N}{\underset{n=1}{\sum }}\overset{M}{\underset{m=1}{\sum }}p\left(x_{n},y_{m}\right)\ln p\left(x_{n}{|y}_{m}\right)$$

where H(X|Y) is the conditional entropy. n and m represent the number of class intervals of X and Y, and $p\left(x_{n}{|y}_{m}\right)$ represents the conditional probability. 

Three rainfall stations each were initially present in the Goesan Dam and Hwacheon Dam watersheds, which was not sufficient to characterize the complete rainfall observation data in this case. This situation was improved by increasing the number of rainfall stations to 8 and 9 in the two watersheds, including rainfall stations but outside the watershed. Radar data at the location of the rainfall stations were also used in the importance ranking of the stations using entropy. If the value of quantitative precipitation estimated using radar data, and the measured precipitation, are in agreement, it indicates the priority ranking of the rainfall station location is identical in the two cases.

The dual polarimetric radars installed at Mt. Gwangdeok (GDK), Mt. Gari (GRS), and Mt. Kwaak (KWK) cover both the Goesan Dam and the Hwacheon Dam watersheds; therefore, it is necessary to identify the radar that is optimal for both watersheds. Regarding radar data, data from GDK, GRS, and KWK, mosaic by maximum value (MMV), and mosaic by averaged value (MAV) were considered in this study. The optimal radar was selected using the five radar datasets and the Spearman rank correlation coefficient of the rainfall observation data. After selecting the optimal radar data for each dam, the locations of the additional rainfall stations in the watershed were obtained using conditional entropy. In addition, the efficacy of an additional rainfall station was evaluated as the decrease in the total entropy when the additional rainfall station was installed, compared to the total entropy under the currently installed rainfall stations. However, if the determined location of the additional rainfall station in the Hwacheon Dam watershed were to fall within the territory of North Korea, then actual installation would not be possible. Therefore, the geographical location of the road network in the watershed, and the corresponding radar data, were comprehensively considered to determine the location of rainfall stations near the road network.

## 3. Study Area and Data

### 3.1. Study Area

The areas included in this study are the Goesan Dam and Hwacheon Dam watersheds located in the Bukhangang River basin. Both dams are located in mountainous areas and the time taken for rainwater inflow to the reservoir after a rainfall event is very short. Therefore, for agile and sensitive response to localized heavy rainfall events, accurate observations of rainfall in the watershed are important; for example, an incident of flood due to a localized heavy rainfall event (6 h with a rainfall intensity of 30.5 mm/h) resulted in damages upstream and downstream of the Goesan Dam on 16 July 2017. In addition, the difference between the observed and predicted rainfall in these watersheds was large, causing difficulties in dam operation.

The watershed of Goesan Dam is 676.7 km^2^ in area, and the total storage capacity and effective storage capacity of the reservoir are 9,714,000 m^3^ and 5,319,000 m^3^, respectively. The design flood level and the design flood are 136.93 m above mean sea level (MSL) and 2711 m^3^/s, respectively; the normal high-water-level is 135.65 m above MSL; and the restricted water level is 134.00 m above MSL. Hwacheon Dam is located in the upstream area of the Bukhangang River basin, the watershed being 3845.5 km^2^ in area; the Paro Lake which is the reservoir of the dam has an area of 32.48 km^2^ at high-water-level, and a total storage capacity and effective storage capacity of 908,908,000 m^3^ and 573,383,000 m^3^, respectively. The design flood level and the design flood are 183.00 m above MSL and 9500 m^3^/s, respectively; the normal high-water-level is 181.00 m above MSL; and the restricted water level is 175.00 m above MSL (http://www.hrfco.go.kr/web/sumunPage/dictionary.do#, https://www.khnp.co.kr/content/220/main.do?mnCd=FN060202, accessed on 17 February 2021).

### 3.2. Data

Radar data on rainfall in Korea can be obtained through weather radar operated by the Korea Meteorological Administration (KMA) and rainfall radar operated by the Ministry of the Environment (http://hrfco.go.kr/web/openapiPage/openApi.do, https://data.kma.go.kr/, accessed on 17 February 2021). Radar data on rainfall for this study were derived from datasets of the GDK and KWK of the KMA and GRS of the Ministry of Environment. In terms of radar data from individual sites, considering an observation radius is in the range of 150–240 km, the entire watershed can be observed even if only one site is used. However, different observation results may be obtained for the same rainfall event depending on the location and operation strategy of each site.

GDK, GRS, and KWK are all S-band dual polarimetric radars. GDK and KWK have a maximum observation radius of 240 km, and GRS has a maximum observation radius of 150 km. The gate size that provides the maximum spatial resolution that can be calculated from radar observation data is 250 m for the KMA radar and 125 m for the Ministry of Environment radar. The temporal resolution is every 5 min for the KMA radar, and every 1 to 5 min for the Ministry of Environment radar. All data for each site are provided in the form of raw radar volume data in Universal Format (UF), and in the file, radar site header information and dual polarimetric radar variables namely reflectivity (DZ, CZ), radial velocity (Vr), differential reflectivity (Z_DR_), differential phase(φDP), specific differential phase(K_DP_), and cross-correlation coefficient (ρHV) are included; for rainfall estimation using the dual polarimetric radar, reflectivity, differential reflectivity, and specific differential phase are mainly used as variables.

The following details regarding the collection of radar rainfall data used in this study may be noted. The spatial resolution of the GDK, GRS, and KWK radars was set to 1 km. The raw radar data are in the form of 3D volume data in UF format, and the results obtained vary depending on the data extraction method. In this study, data were extracted using the Constant Altitude PPI (CAPPI) method. CAPPI extracts data related to a constant height of interest by storing multiple layers of PPI observation data for representation.

Radar data for each site were converted into rainfall intensity using the JPOLE algorithm [21], which is a radar rainfall estimation technique that uses dual polarimetric radar variables (reflectivity, differential reflectivity, and specific differential phase). 

**Figure 1:**
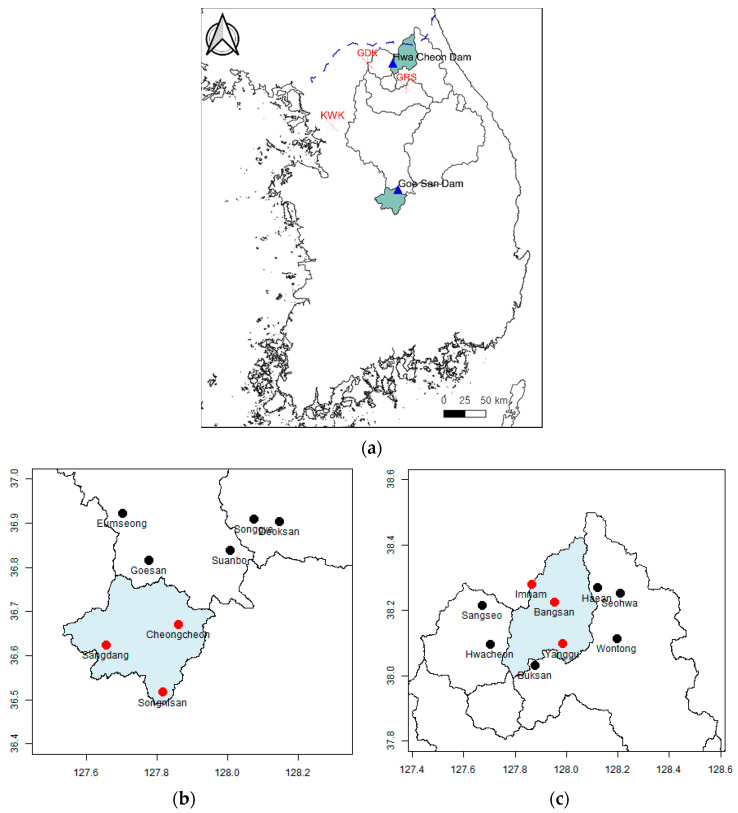
The location of radar equipment and gauged stations. (**a**) Study area and the location of radar. (**b**) Gauged stations in Goesan Dam watershed. (**c**) Gauged stations in Hwacheon Dam watershed.

Regarding observed rainfall data, the rainfall per minute measured by the disaster prevention weather monitoring system of KMA was reorganized into units of 5 min, for use in this study. The study period is from 2018 to 2019, and observation data of 106 days, which is the total number of days of heavy rainfall events during the period, were used. Figure 1 shows the radar observation system and the locations of the rainfall stations in the Goesan Dam and Hwacheon Dam watersheds.

### 3.3. Standard Node Link

The standard node link is an electronic traffic map developed for more efficient management of the collection and provision of traffic information, to improve the accessibility of traffic information [22]. The standard node link contains information such as the class of the road, speed limit, and intersections on the road on which vehicles travel through the nodes and links. In this study, it was used to identify the geographical location of roads in the watershed. As of September 2019, the Hwacheon Dam watershed is represented by 334 links and 32,911 pieces of location information and the Goesan Dam watershed is represented by 605 links and 63,824 pieces of location information.

**Figure 2:**
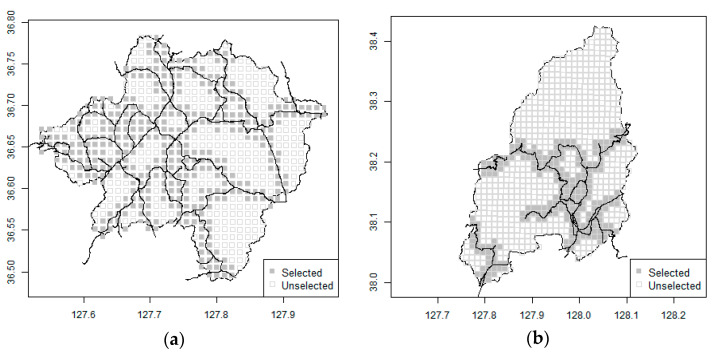
Potential grid nearby the road. (**a**) Goesan. (**b**) Hwacheon.

If rainfall stations are installed close to the road, cost for additional installation and maintenance can be reduced. Considering ease of access, only locations within radar grids around the road were selected (Figure 2). In the Goesan Dam watershed, 419 of the 691 grids or 60.6% of the grids, are close to the road. For the Hwacheon Dam watershed, 268 out of the 938 grids were selected, representing only 28.6% of the grids.

## 4. Results

### 4.1. Optimal Radar Selection

**Figure 3:**
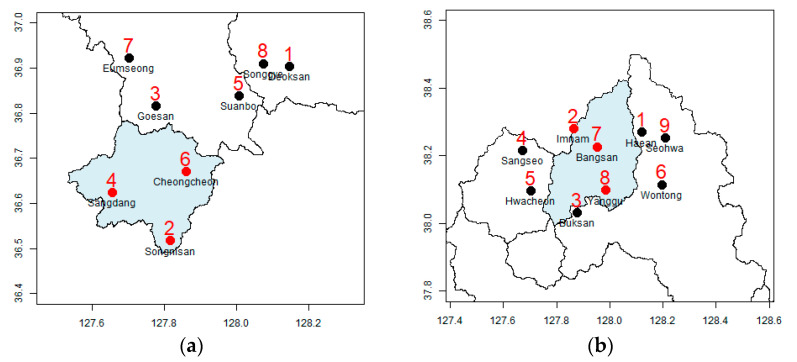
Result of conditional entropy using gauged data. (**a**) Goesan. (**b**) Hwacheon.

To select the optimal radar for determining the rainfall pattern of the watershed in the study area, the importance of rainfall stations, including those in the vicinity of the study area, was calculated using conditional entropy. The importance of radar rainfall estimation at the location of the rainfall station was also calculated using conditional entropy. There are five radar rainfall estimations considered in the study, and the results are shown in Table 1 and Figure 3. Based on observed rainfall data, the rainfall stations in the Goesan Dam watershed in the order of their rank of importance are: Deoksan, Songnisan, Goesan, Sangdang, Suanbo, Cheongcheon, Eumseong, and Songgye. For the Hwacheon Dam watershed, the rank order is Haean, Imnam, Buksan, Sangseo, Hwacheon, Wontong, Bangsan, Yanggu, and Seohwa. The importance ranks of the corresponding rainfall station locations for rainfall estimation by radar were different from the above rank order. The correlation between observed rainfall data and rainfall data estimated using radar was analyzed using the Spearman rank correlation coefficient. The correlation was not high except for the correlation between the mean reflectivity mosaic of the observations for the Hwacheon Dam watershed and KWK radar data.

**Figure 4:**
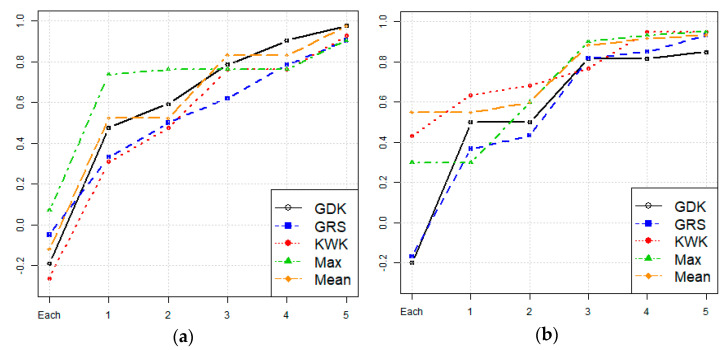
Result of Spearman correlation considering gauged network. (**a**) Goesan. (**b**) Hwacheon.

This study redesigns the rainfall monitoring network by adding additional rainfall stations to the network already installed and in active operation. Assuming the installation of additional rainfall stations in the watershed, Table 2 and Figure 4 show the Spearman rank correlation between the ranking of rainfall stations based on the number of rainfall stations, and the ranking based on the radar network. Both Goesan Dam and Hwacheon Dam showed an increase in the Spearman correlation coefficient after installing important ground rainfall stations. In the case of Goesan Dam, the Spearman correlation coefficient was 0.738 based on the radar maximum ensemble, when the rainfall station was selected by the radar after one additional rainfall station was already selected. In the case of Hwacheon Dam, the Spearman correlation coefficient was 0.633 when the rainfall station was directly selected by the KWK radar. This indicates that, compared to the rainfall estimation obtained from radar data alone, there is better agreement between the actual rainfall uncertainty and the uncertainty in the radar rainfall estimation when the location of the rainfall station in actual operation is used.

### 4.2. Selection of Additional Rainfall Stations

The conditional entropy of the entire watershed was calculated using radar data for each selected watershed. The maximum reflectivity mosaic was used for the Goesan Dam watershed, and KWK was used for the Hwacheon Dam watershed. Table 3 shows the results based on the number of additional rainfall stations, based on the conditional entropy. Here, ER is the entropy reduction ratio following the installation of the additional rainfall station, and is calculated by the following equation:

(3) $$\mathrm{ER}=1-\sum_{i=j+1}^{N}H\left(X_{i}|Y_{m+j}\right)/\sum_{i=1}^{N}H(X_{i}|Y_{m})\times 100$$

where $\sum_{i=1}^{N}H(X_{i}|Y_{m})$ is the cumulative conditional entropy of radar rainfall estimation when m number of rainfall stations were selected in the watershed, and $\sum_{i=j+1}^{N}H(X_{i}|Y_{m+j})$ represents the cumulative conditional entropy of radar rainfall estimation when j number of rainfall stations were additionally installed.

**Figure 5:**
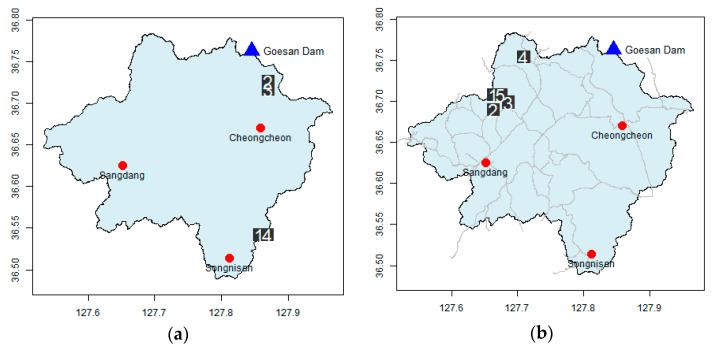
Results of the location of additional rainfall stations in the Goesan Dam watershed. (**a**) Proposed gauge sequence. (**b**) Proposed gauge sequence considering road.

**Figure 6:**
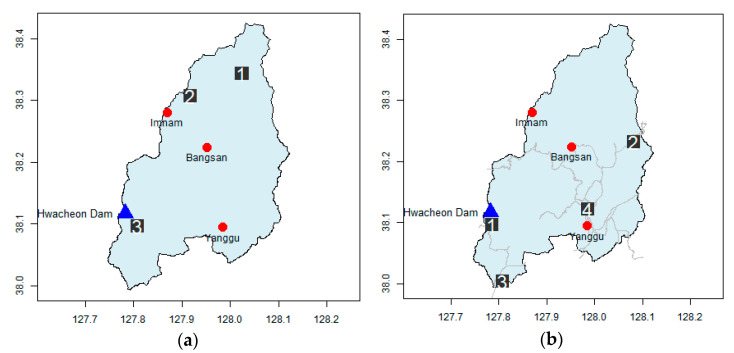
Results of the location of additional rainfall stations in the Hwacheon Dam watershed. (**a**) Proposed gauge sequence. (**b**) Proposed gauge sequence considering road.

For example, in the Goesan Dam watershed, if four additional rainfall stations are installed, the uncertainty in rainfall is reduced by 70.0% from the value obtained during the operation of three rainfall stations. In the Hwacheon Dam watershed, if three additional rainfall stations are installed, the rainfall uncertainty reduces by 67.9% from the value obtained during the operation of the original three rainfall stations alone. However, the installation of the additional rainfall stations in identified locations is difficult in practice because the locations potentially important for the Goesan Dam watershed are deep in the middle of Gunjasan and Songnisan, and locations 1 and 2 which are potentially important for the Hwacheon Dam fall under North Korean territory (Figure 5 and Figure 6).

### 4.3. Selection of Location of Additional Rainfall Stations Considering Road Network

**Figure 7:**
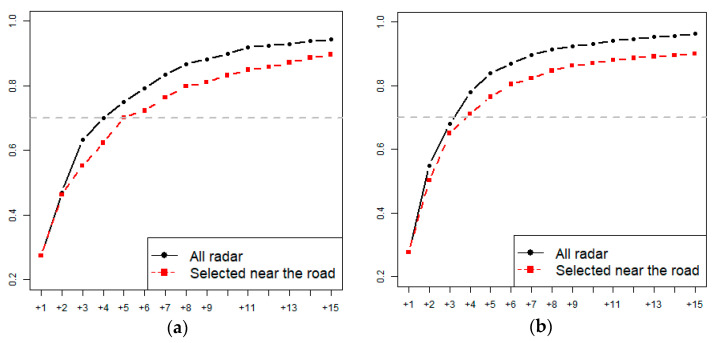
Comparison of ER values of additional gauging sites. (**a**) Goesan. (**b**) Hwacheon.

To ensure efficient operation of the additional rainfall stations selected, stations close to the road were selected, by performing masking through incorporation of the geographic information of road network data. The results of the analysis are presented in Table 4 and Figure 7. 

Compared to Table 3, which shows the results for additional stations selected using radar data, the efficiency is slightly lower, but lower installation cost and easy maintenance are the advantages in this case. If the entropy reduction is set to approximately 70%, five additional rainfall stations need to be installed for the Goesan Dam watershed (70.1%) and four additional rainfall stations for the Hwacheon Dam watershed (71.1%). The number of additional rainfall stations is increased by one compared to the method described in Section 4.2, but if the installation of the additional station is economically viable, this method that uses the road network for the selection of additional rainfall stations would be appropriate.

Figure 5 shows a detailed depiction of the locations of the additional rainfall stations in the Goesan Dam watershed. The installation of four additional rainfall stations in the Goesan Dam watershed reduced the uncertainty by 70.0%; the east–south and east–west sides are selected as major locations. In Songnisan (1058 m), located in the southwest of the watershed, and Gunjasan (948 m), located in the east–west side—both in mountainous areas with elevations higher than the surroundings—the uncertainty in rainfall estimation was high. If five rainfall stations are added limiting their locations to radar grids close to the road, uncertainty can be reduced by 70.1%; Jwagusan (657 m) located on the northwest side and an adjacent natural recreational forest are selected.

Figure 6 shows the spatial characteristics of the additional rainfall stations in the Hwacheon Dam watershed. Adding three rainfall stations reduces the uncertainty by 67.9%, and the south and north sides of the watershed are chosen as the main locations for the rainfall stations. Since the northern part of the Hwacheon Dam watershed falls under North Korean territory, rainfall stations cannot be installed there. If four rainfall stations are added close to the roads within the national territory, the uncertainty is reduced by 71.1%, and uniform distribution of stations in all directions is achieved. The location with the highest uncertainty in rainfall in South Korean territory is the area around Paro Lake.

## 5. Conclusions

In this study, the conditional entropy for observed rainfall data and that for rainfall estimation data from radar measurements were compared to identify locations of additional rainfall stations that would enable effective reduction in the uncertainty in quantitative rainfall estimation. Radar data for rainfall estimation appropriate for the watershed studied were selected based on the correlation between the importance values for the rainfall stations and the importance values for the radar-based rainfall estimation at the locations of the rainfall stations. In the Goesan Dam watershed, the correlation coefficient of the maximum ensemble was the highest at 0.738; in the Hwacheon Dam watershed, the KWK radar showed the highest correlation at 0.633. Next, the conditional entropy method was used for rainfall estimation based on radar measurements at the locations of the rainfall stations. Qualitative evaluation revealed a reduction in the entropy rate due to the addition of a rainfall station; the uncertainty in the Goesan Dam watershed reduced by 70.0% when four additional rainfall stations were installed, and by 67.9% in the Hwacheon Dam watershed when three additional rainfall stations were installed. In addition, when only the radar grids close to the road are selected by incorporating the geographical location of roads in the watershed, the economic viability in terms of installation and operation can be improved. When five additional rainfall stations were installed in the Goesan Dam watershed using the proposed method, the entropy was reduced by 70.1% while four additional rainfall stations installed in the Hwacheon Dam watershed reduced the entropy by 71.1%. Compared to the design method of adding stations to the rainfall monitoring network based on rainfall estimation of the entire watershed using radar measurements, the uncertainty reduction is smaller in this case; however, the method is considered suitable in terms of ease of maintenance and lesser cost of installation and operation.

## Figures and Tables

**Table 1:** Result of conditional entropy using gauged and radar data.

Region		Gauged	Radar				
			GDK	GRS	KWK	MMV	MAV
Goesan	Deoksan	1	8	5	5	5	7
	Songnisan	2	2	3	4	3	1
	Goesan	3	5	2	7	2	6
	Sangdang	4	4	6	1	4	2
	Suanbo	5	6	7	8	8	8
	Cheongcheon	6	3	8	6	7	5
	Eumseong	7	1	4	3	6	4
	Songgye	8	7	1	2	1	3
	Spearman	-	−0.191	−0.048	−0.262	0.071	−0.119
Hwacheon	Haean	1	9	7	5	1	1
	Imnam	2	1	2	2	5	3
	Buksan	3	7	9	4	8	8
	Sangseo	4	3	5	8	3	5
	Hwacheon	5	5	4	1	6	2
	Wontong	6	6	1	3	4	4
	Bangsan	7	8	8	7	9	9
	Yanggu	8	2	6	9	2	7
	Seohwa	9	4	3	6	7	6
	Spearman	-	−0.200	−0.167	0.433	0.300	0.550

**Table 2:** Result of Spearman correlation considergind gauged network.

Region	Radar	Spearman					
		Each	1	2	3	4	5
Goesan	GDK	−0.190	0.476	0.595	0.786	0.905	0.976
	GRS	−0.048	0.333	0.500	0.619	0.786	0.905
	KWK	−0.262	0.310	0.476	0.762	0.762	0.929
	MMV	0.071	0.738	0.762	0.762	0.762	0.905
	MAV	−0.119	0.524	0.524	0.833	0.833	0.976
Hwacheon	GDK	−0.200	0.500	0.500	0.817	0.817	0.850
	GRS	−0.167	0.367	0.433	0.817	0.850	0.933
	KWK	0.433	0.633	0.683	0.767	0.950	0.950
	MMV	0.300	0.300	0.600	0.900	0.933	0.950
	MAV	0.550	0.550	0.600	0.883	0.917	0.933

**Table 3:** Result of conditional entropy in additional gauged stations by watershed.

Only Radar	Goesan			Hwacheon		
Additional Station	Entropy	Sum of Entropy	ER	Entropy	Sum of Entropy	ER
+1	1.003	142.428	27.4%	0.886	312.685	27.7%
+2	0.818	104.134	46.9%	0.554	194.638	55.0%
+3	0.626	72.011	63.3%	0.426	138.662	67.9%
+4	0.535	58.841	70.0%	0.316	95.064	78.0%
+5	0.434	48.838	75.1%	0.251	69.661	83.9%
+6	0.328	40.869	79.2%	0.223	56.418	86.9%
+7	0.273	32.532	83.4%	0.183	44.462	89.7%
+8	0.198	26.027	86.7%	0.143	37.088	91.4%
+9	0.160	22.974	88.3%	0.126	32.620	92.5%
+10	0.140	19.950	89.8%	0.119	29.698	93.1%
+11	0.117	15.782	92.0%	0.102	25.341	94.1%
+12	0.090	14.906	92.4%	0.091	23.007	94.7%
+13	0.082	13.792	93.0%	0.084	20.246	95.3%
+14	0.067	12.111	93.8%	0.077	18.806	95.6%
+15	0.058	11.069	94.4%	0.069	16.085	96.3%

**Table 4:** Result of conditional entropy in additional gauged stations considered road information by watershed.

Add Road	Goesan			Hwacheon		
Additional Station	Entropy	Sum of Entropy	ER	Entropy	Sum of Entropy	ER
+1	0.726	142.428	27.4%	0.675	312.685	27.7%
+2	0.539	105.294	46.3%	0.466	215.053	50.3%
+3	0.334	87.798	55.3%	0.352	151.223	65.0%
+4	0.294	73.890	62.3%	0.272	124.986	71.1%
+5	0.271	58.624	70.1%	0.187	101.490	76.5%
+6	0.182	54.452	72.2%	0.150	84.535	80.4%
+7	0.168	46.248	76.4%	0.136	76.399	82.3%
+8	0.141	39.626	79.8%	0.118	66.093	84.7%
+9	0.128	37.024	81.1%	0.092	59.234	86.3%
+10	0.102	32.903	83.2%	0.071	55.694	87.1%
+11	0.091	29.638	84.9%	0.066	51.660	88.1%
+12	0.085	27.985	85.7%	0.062	48.868	88.7%
+13	0.076	25.127	87.2%	0.051	46.751	89.2%
+14	0.072	22.316	88.6%	0.050	45.177	89.5%
+15	0.064	20.381	89.6%	0.047	43.247	90.0%

## Data Availability

The data presented in this study are available in http://hrfco.go.kr/web/openapiPage/openApi.do and https://data.kma.go.kr/, accessed on 17 February 2021.
